# Enhanced cell proliferation and osteogenic differentiation in electrospun PLGA/hydroxyapatite nanofibre scaffolds incorporated with graphene oxide

**DOI:** 10.1371/journal.pone.0188352

**Published:** 2017-11-29

**Authors:** Chuan Fu, Haotian Bai, Jiaqi Zhu, Zhihao Niu, Yu Wang, Jianan Li, Xiaoyu Yang, Yunshen Bai

**Affiliations:** 1 Department of Orthopedic Surgery, the Second Hospital of Jilin University, Changchun, Jilin, P. R. China; 2 Department of Hepatobiliary Surgery, the Third Center Hospital of Tianjin, Tianjin, Tianjin, P. R. China; 3 Key Laboratory of Polymer Ecomaterials, Changchun Institute of Applied Chemistry, Chinese Academy of Sciences, Changchun, Jilin, P. R. China; Michigan Technological University, UNITED STATES

## Abstract

One of the goals of bone tissue engineering is to mimic native ECM in architecture and function, creating scaffolds with excellent biocompatibility, osteoinductive ability and mechanical properties. The aim of this study was to fabricate nanofibrous matrices by electrospinning a blend of poly (L-lactic-co-glycolic acid) (PLGA), hydroxyapatite (HA), and grapheme oxide (GO) as a favourable platform for bone tissue engineering. The morphology, biocompatibility, mechanical properties, and biological activity of all nanofibrous matrices were compared. The data indicate that the hydrophilicity and protein adsorption rate of the fabricated matrices were significantly increased by blending with a small amount of HA and GO. Furthermore, GO significantly boosted the tensile strength of the nanofibrous matrices, and the PLGA/GO/HA nanofibrous matrices can serve as mechanically stable scaffolds for cell growth. For further test in vitro, MC3T3-E1 cells were cultured on the PLGA/HA/GO nanofbrous matrices to observe various cellular activities and cell mineralization. The results indicated that the PLGA/GO/HA nanofibrous matrices significantly enhanced adhesion, and proliferation in MCET3-E1 cells and functionally promoted alkaline phosphatase (ALP) activity, the osteogenesis-related gene expression and mineral deposition. Therefore, the PLGA/HA/GO composite nanofibres are excellent and versatile scaffolds for applications in bone tissue regeneration.

## Introduction

Biodegradable polymeric scaffolds for bone reconstruction have received significant attention because of the limitations of bone tissue regeneration potential and current treatments [1, 2]. Ideal bone tissue scaffolds should have a suitable structure to mimic temporary extracellular matrix (ECM), which can control cellular behaviours and provide appropriate microenvironments [3]. Therefore, over the past few years, scaffolds with various architectural configurations and geometries have been designed and fabricated to mimic ECM using a variety of methods and materials, such as electrospinning, melt extrusion, rapid prototyping and solvent evaporation [4–7]. Of these, electrospinning has attracted interest as a simple and effective method because electrospun scaffolds are highly porous, and have a high specific surface area and ECM-like nanotopography. Many studies reported that synthetic biodegradable polymers such as poly (lactic-co-glycolicacid) (PLGA) have been used to fabricate nanofibrous scaffolds by electrospinning for bone tissue engineering, alone or combined with other biomaterials [8–10].

Over the past decade, PLGA, hydroxyapatite (HA) and/or their combination have been used extensively as artificial scaffolds for bone tissue engineering [11–13]. PLGA is a biocompatible polymer that is extensively used for biomedical application due to its excellent biocompatibility, biodegradability, and degradation rate can be adjusted by altering the ratio of lactic to glycolic acids [14, 15]. However, hydrophobic surfaces, unsatisfactory mechanical properties and a lack of bioactivity seriously limit the biological applications of electrospun PLGA scaffolds. To address these issues, various materials have been incorporated into PLGA-based scaffolds [15–17]. Among these materials, hydroxyapatite, an effective component for biomimetic materials, has been widely used in bone tissue engineering because of its good biocompatibility and osteoconductivity. Many studies have reported that PLGA/HA composites have good biocompatibility and provide an environment that can markedly improve the osteogenic differentiation and mineralization of cells [11, 18]. Our group is also committed to the study of PLGA/HA composite scaffolds for the bone repair. However, according to some experimental studies, HA exhibits poor mechanical properties such as intrinsic brittleness, low fracture toughness and low wear resistance, and HA by itself possesses limited osteoinductive ability, all of which seriously limit the biological applications of PLGA/HA composite scaffolds [18–20]. Therefore to improve the mechanical properties and bioactivity of the PLGA/HA composite scaffolds, various methods have been tried in the past.

Graphene, a single two-dimensional layer of carbon, and its derivatives have been applied in many field, including gene/drug delivery, cancer photothermal therapy and tissue engineering, because of their unique physicochemical characteristics including optical, electrical and thermal conductivity, and a high surface to volume ratio [21–24]. Graphene oxide (GO) is the oxidized form of graphene and has many hydrophilic functional groups, such as hydroxyl and carboxyl, which confer a higher dispersibility in aqueous solutions and better hydrophilicity than graphene [25]. Recently, it was reported that the biocompatibility of HA can be significantly improved by the incorporation of GO [26]. Furthermore, the incorporation of GO into polymeric scaffolds has been reported to enhance cell adhesion, proliferation and osteogenic differentiation. For example, Lou et al. found that the incorporation of GO into PLGA nanofibres could facilitate the proliferation and osteogenic differentiation of mouse mesenchymal stem cells [27]. Therefore, GO is one of the most promising bio-building blocks for scaffold substrates to promote cellular behaviour. We speculate that blending PLGA/HA composite scaffolds with GO can improve the mechanical properties and osteoinductive ability of the scaffolds efficiently, thereby making them more suitable for bone tissue engineering applications.

In this study, to obtain an ideal scaffold with improved mechanical and biological properties, GO-impregnated biomimetic matrices composed of PLGA and HA (PLGA/GO/HA) were fabricated via an electrospinning process. Through scanning electron microscope (SEM), X-ray diffraction (XRD), contact angle measurements, and materials testing, the physicochemical and mechanical properties of the nanofibrous matrices were characterized. Furthermore, the PLGA/GO/HA nanofibrous matrices were then subjected to cell culture (MC3T3-E1 cell) to evaluate the effect of GO and HA on cell behaviour, including adhesion, proliferation, and differentiation.

## Experimental

### Materials

PLGA [molecular weight (MW) = 150000, PLA:PGA = 75:25 (mol/mol)] and needle-like HA nanocrystals (HA) of 20−30 nm in diameter and 100−200 nm in length were synthesized by Changchun Institute of Applied Chemistry, Chinese Academy of Sciences (CIAC, China). GO was purchased from Chengdu Organic Chemicals Co. Ltd, China (thickness: 0.55–1.2nm, diameter: 0.5–3μm). Bovine serum albumin (BSA) was obtained from Beijing Solarbio Science & Technology Co., Ltd. 1,1, 1, 3, 3,3-hexafluoroisopropanol (HFIP), 3-(4,5-dimethyl-2-thiazolyl)-2, 5-diphenyl-2- H-tetrazolium bromide (MTT), and BCA protein assay kit were purchased from Sigma-Aldrich (USA). The reagents for cell experiments were purchased from Gibco (USA).

### Fabrication of PLGA/GO/HA fibrous matrices

PLGA/GO/HA nanofibrous matrices were prepared by electrospinning the admixture of PLGA and HA blended with GO [28]. Briefly, 0.2 g HA was dispersed in 1mL HFIP and 1.8 g PLGA was dissolved in 9 mL HFIP. Then, the HA suspension was dropped into PLGA solution and subsequently stirred at 500 rpm for 12 h. The PLGA/HA solution was then doped with 0.04g GO and homogenized for 2 h by ultrasonication. The total solids content of HA and GO was 10% and 2% (w/w), respectively. The mixture solution of PLGA, HA and GO was then loaded into a syringe. After that, the mixture solution of PLGA, HA and GO was directly electrospun onto the aluminum foil-covered collector (applied voltage: 20 kV, flow rate of the solution: 1 mL/h, air gap distance: 20 cm; injection needle diameter: 0.61 mm). To remove the residual solvent, the PLGA/GO/HA nanofibrous matrices were completely dried under vacuum at room temperature for 1 day.

### Characterization

Scanning electron microscope (SEM, XL30 ESEM-FEG, FEI) was used to observe the morphology of the nanofibrous matrices. X-ray diffraction (XRD, D8 ADVANCE, Germany) was used to determine the chemical properties of the nanofibous matrices. To analyse the surface hydrophobicity, the water contact angles of the nanofibrous matrices were measured using a contact angle measurement system (VAC2000, 135 AST). The mechanical properties of the nanofibrous matrices were determined at 20°C with a universal mechanical testing machine (Instron 1121, UK) using nanofibrous matrices with width of 10 mm and initial length of 30 mm.

### Protein adsorption capacity of PGA/GO/HA nanofibrous matrices

BSA was selected as a model protein to determine the adsorption efficiencies of synthesized nanofibrous matrices. The matrices were tailored into 15mm discs, fitted into a tube, and incubated with 10 mL of BSA solution (10 mL, 2 mg/mL) under stirring at 150 rpm for 24 h. The adsorbed BSA concentration was determined through the decrease of the concentration of BSA within the samples using a BCA kit. Furthermore, Rhodamine B labelled BSA (Sigma) solution (1 mg/mL) was incubated on the matrices for 2 h. Then the samples were rinsed in PBS 3 times and mounted for visualization with a fluorescence microscope (TE2000-U, Nikon).

### Cell spreading, attachment, and proliferation assays

MC3T3-E1 cells (ATCC subclone 14) were cultured in high-glucose DMEM (containing 10% foetal bovine serum, 100 U/mL penicillin, and 100 mg/mL streptomycin). The culture conditions were a humidity controlled environment under 5% CO_2_ and 95% air at 37°C. For cell seeding, the nanofibrous matrices (PLGA, PLGA/GO, PLGA/HA, PLGA/GO/HA) were sterilized by immersing them in 70% alcohol for 30 min, washed twice with sterile PBS, and seeded with MC3T3-E1 cells at 3.5 × 10^4^ cells· mL^−1^· well^−1^ in a 24-well plate. The medium was changed every 2 d. After 1, 4 and 7 d of culture, the medium was replaced by MTT assay reagents. Briefly, 20 μL of 100 mL of MTT (5 g L^−1^ in PBS) was added to each well, followed by incubation at 37°C cell incubator for 4 h. Then, the medium was removed and the formed formazan crystals were dissolved by the addition of 800 μL acidified isopropanol (0.2 mL of 0.04 N hydrochloric acid (HCl) in 10 mL of isopropanol). After the incubation period, the 200 mL of the solution in each well was pipetted out into another 96 well plate for the absorbance measurement at 540 nm on the multifunctional microplate scanner (Infinite M200, TECAN).

For the cell spreading and attachment examination, after the stipulated time period (1, 3 and 7 days), non-adherent cells were washed away with PBS, while adherent cells were fixed with 4.0% paraformaldehyde for 30 min. After being washed 3 times with PBS, samples was hatched with fluorescein isothiocyanate (FITC) (0.5 mg·mL^−1^ in DMSO) for 5 min and rinsed repeatedly. Then, the cell nucleus was stained using 4′,6-diamidino-2-phenylindole (DAPI; Sigma-Aldrich, USA). Finally, the samples were observed by observed under a fluorescence microscope (TE2000-U, Nikon).

### Alkaline phosphate activity assay

ALP activity was determined after culturing for 7 and 14 days by quantitation of the enzyme activity. Briefly, the medium from each well was carefully removed, and the cells were washed three times with PBS and split by repeated freezing and thawing. Fifty microlitres of pNPP solution was placed in each well away from light and maintained at 37°C for 30 min. The reaction was terminated with 3M NaOH and the OD values at 405 nm were determined via a full wavelength reader. The relative ALP activity was represented as the average OD values.

### Osteogenic genes expression

The MC3T3-E1 cells cultured on various nanofibrous matrices for 7 days were also collected for the evaluation of the osteogenesis-related genes expression. Total RNA was extracted using TRIzol Reagent (Invitrogen) according to the manufacturer’s protocol. The total RNA concentration and purity were detected by a Nanodrop assay (Tecan M200), and the first strand cDNA was synthesized by reverse transcriptase as described in theM-MLV manual (Promega). The expression of osteogenic markers was quantified by qPCR SYBRGreen Mix Kit (TaKaRa). The primer sequences specific for the target gene including anti-runt-related transcription factor 2(RUNX2), osteopontin (OPN) and glyceraldehyde-3-phosphate dehydrogenase (GAPDH) used for qRT-PCR are listed in Table 1. The specificities of the listed oligonucleotides were checked by BLASTN® (Basic Local Alignment Search Tool) against the mouse RefSeq RNA database at NCBI. The qPCR amplification was done as follows: initial denaturation at 95°C for 10min, followed by 40 cycles at 95°C for 30 s, 58°C for 1 min, 72°C for 1 min. The comparative threshold cycle method was used to analyse the Q-PCR results using iCycleriQ Detection System software with GAPDH as the reference gene. All results were quantified using the ΔΔCt relative quantification method.

**Table 1:** List of genes and primer nucleotide sequences.

Gene	Forward primer sequence	Reverse primer sequence
**RUNX 2**	5-GCCCTCATCCTTCACTCCAAG-3′	5-GGTCAGTCAGTGCCTTTCCTC-3′
**OPN**	5-TCAGGACAACAACGGAAAGGG-3′	5-GGAACTTGCTTGACTATCGATCAC-3′
**GAPDH**	5-CAACCTGGTCCTCAGTGTAGC-3′	5-CGTGCCGCCTGGAGAAACCTGCC-3′

### Mineralization

Alizarin red S (ARS) staining was performed to assess the calcium deposition by MC3T3-E1 cells cultured on the nanofibrous matrices for 14 and 21 days. All nanofibrous matrices were seeded with MC3T3-E1 cells at 2×10^4^ cells/mL density in 24-well plates to evaluate cell mineralization. At every specific time point, the cells were washed with PBS and fixed with 4% paraformaldehyde in PBS at room temperature for 15 min. After being rinsed with PBS, the cells were stained with Alizarin Red S solution (50 mM) at 37°C for 20 min and washed three times with PBS. After the removal of Alizarin Red S solution, the cell/ matrices samples were observed under a light microscope. Calcium quantification was measured using cetylpyridinium chloride (CPC) treatment. The ARS-stained samples were treated with 1 mL of 10% CPC solution for 1h to desorb calcium ions, and the absorbance of the collected dye was read at 540 nm in a multifunctional microplate scanner. Furthermore, after 21 d of culture, cell/matrices samples were washed with PBS and fixed with 4% paraformaldehyde for 3 h at room temperature. Samples were then washed with PBS three times, dehydrated through a graded series of ethanol (50%, 70%, 80%, 90%, 95%, and 100%) and dried under vacuum. Finally, the samples were coated with gold and observed by SEM.

### Statistical analysis

All quantitative data were analysed with OriginPro 8.0 (Origin Lab Corporation, USA) and presented as the mean ± standard deviation. Statistical differences were performed by one-way analysis of variance (ANOVA). A value of p < 0.05 was considered to be significant.

## Results and discussion

### Characterization of PLGA/GO/HA nanofibrous matrices

**Fig 1:**
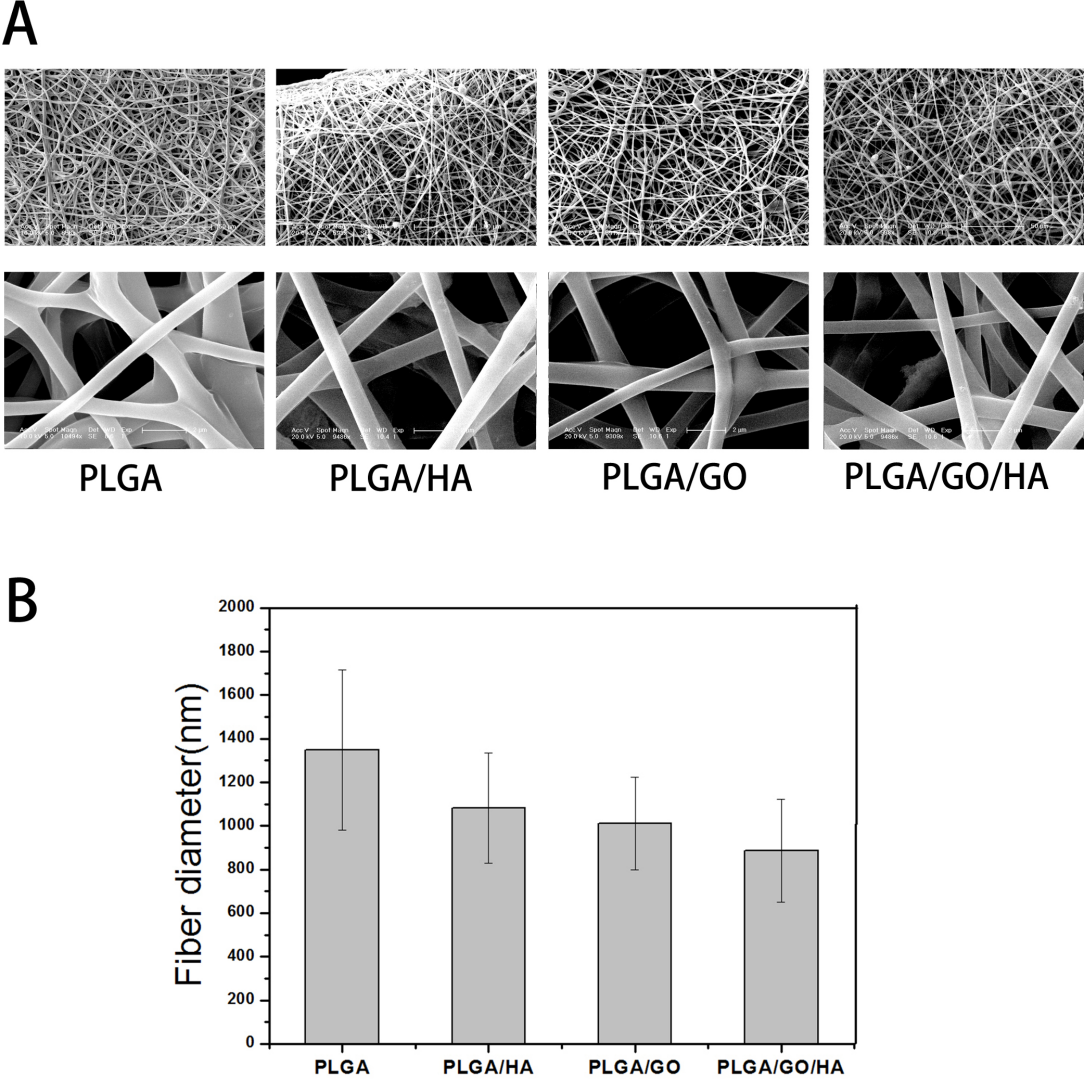
Surface morphological images (A) and average diameter (B) of PLGA, PLGA/HA, PLGA/GO, and PLGA/GO/HA nanofibrous matrices.

PLGA/GO/HA nanofibrous matrices were prepared by electrospinning the admixture of PLGA and HA blended with GO. As shown in Fig 1, uniform and smooth nanofibres with porous three-dimensional structures were fabricated from PLGA by electrospinning. The PLGA nanofibres kept a wide distribution range with a mean diameter of 1347 ± 368 nm. After GO, HA or both were added into the PLGA solution, the diameter of nanofibres showed a slight downward trend (p>0.05), the mean diameter of PLGA nanofibres decreased from 1347 ± 368 to 1009 ± 212, 1082 ± 252 and 885 ± 235 nm. The decrease of fibre diameter is probably due to the change of the solution conductivity and viscosity caused by the GO and HA. The porous structure and the diameter of nanofibrous matrices are similar to the topological structure, and therefore would be favourable for nutrient waste exchange and cell growth. Furthermore, we found that the surface roughness was found on PLGA/HA nanofibres because of the incorporation of HA crystals. However, after GO was added into the PLGA nanofibres, the nanofibres with smooth surfaces could not see GO on the surface of the nanofibres. This phenomenon can be explained partly by the fact that GO nanosheets might be embedded in the nanofibres and aligned along the axial direction of shape anisotropy of regular shapes in nanofibres similar to the literature reported by Lee [14].

**Fig 2:**
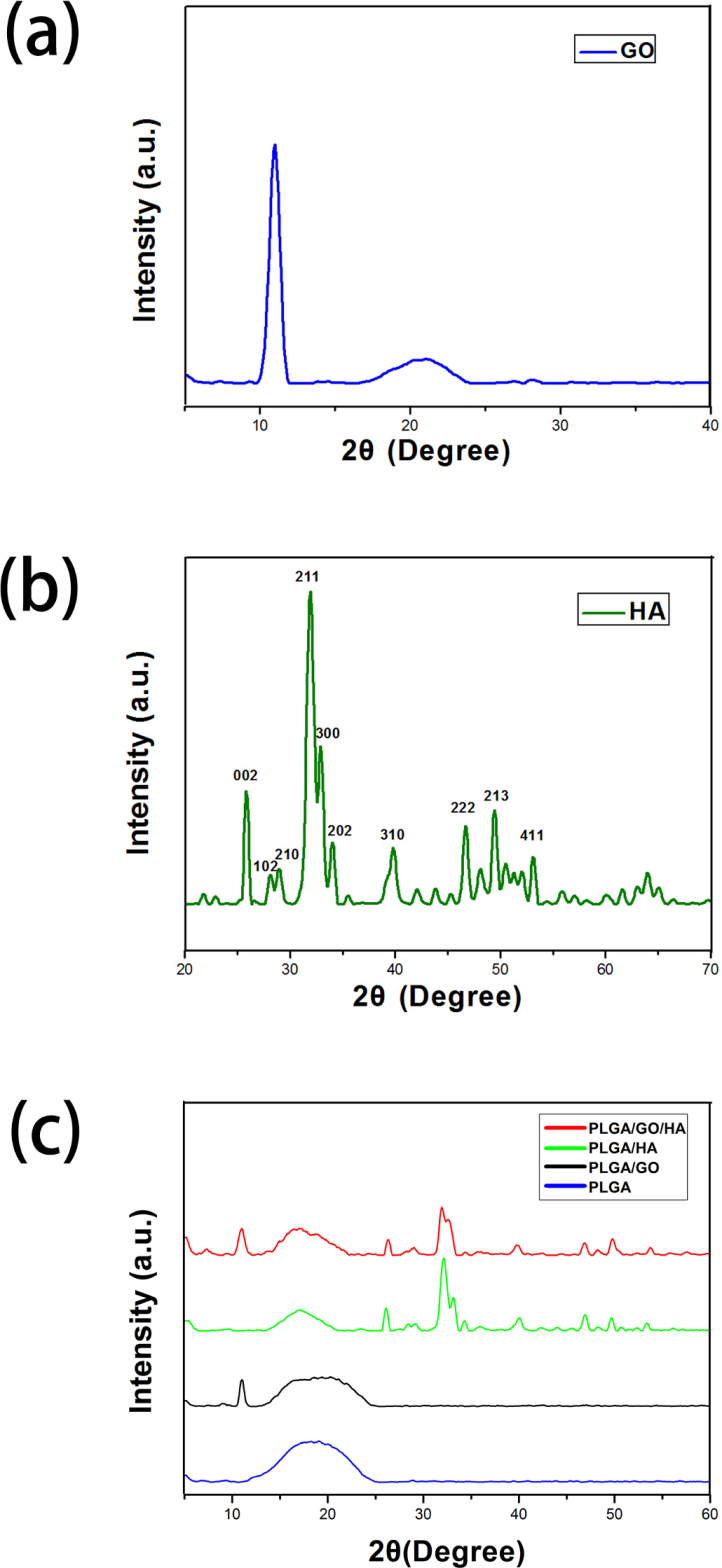
XRD patterns of powdery GO (a) and HA (b), and PLGA, PLA/HA, PLGA/GO and PLGA/GO/HA nanofibrous matrices (c).

The XRD patterns of the PLGA, PLGA/HA, PALGA/GO and PLGA/GO/HA nanofibrous matrices were also collected to determine the surface chemical properties of the nanofibrous matrices. As shown in Fig 2, the characteristic diffractions of (002), (102), (211), (300), (202), (310), (222), (213), and (411) of HA are found in the XRD patterns of PLGA/HA and PLGA/GO/HA nanofibrous matrices. In contrast, PLGA and PLGA/GO nanofibrous matrices did not show any diffraction peak for HA. Furthermore, as shown in Fig 2, the GO pattern shows a characteristic peak at 2θ≈11°, corresponding to an interlayer spacing of 0.79 nm, which is the typical separation of the layered GO. The XRD patterns of PLGA/GO and PLGA/GO/HA nanofibrous matrices exhibited a similar diffraction peak at 2θ≈11°. The above result indicated that HA and GO were exposed on the microcarrier surface.

**Fig 3:**
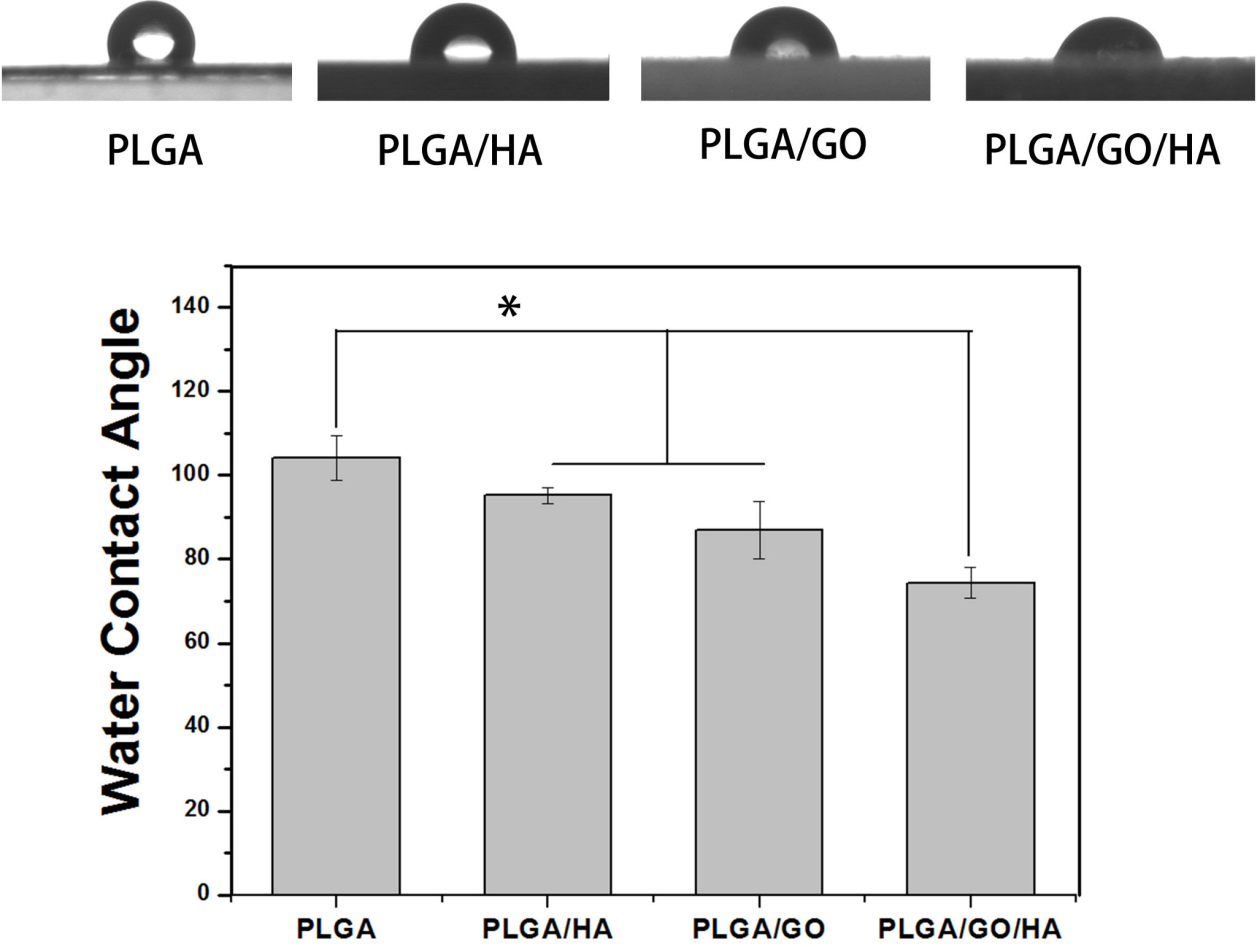
Water contact angle measurement for the PLGA、PLGA/HA、PLGA/GO and PLGA/GO/HA nanofibrous matrices. (n = 5;* p < 0.05).

The hydrophilicity of the materials plays an important role in interacting with cells. As shown in Fig 3, the water contact angle was 104.1 ± 5.2° for the PLGA nanofibrous matrices, 86.9 ± 6.9° for the PLGA/GO nanofibrous matrices, 95.2 ± 1.9° for the PLGA/HA nanofibrous matrices, and 74.4 ± 3.5° for the PLGA/GO/HA nanofibrous matrices. The water contact angles of the PLGA/HA samples were obviously decreased compared with those of the pure PLGA/HA samples due to the exposure of HA on the surface. Doping with graphene oxide further increased hydrophilicity and reduced the water contact angle to 86.9° ± 6.9° because of the presence of hydrophilic OH, C-O-C and COOH groups on the GO surface. Among the nanofibrous matrices, the lowest contact angle was obtained for the PLGA/GO/HA nanofibrous matrices (p<0.05), indicating that HA and GO could change the surface properties of PLGA nanofibrous matrices. The PLGA/GO/HA nanofibrous matrices were expected to show better affinity for cells.

### Protein absorptivity

**Fig 4:**
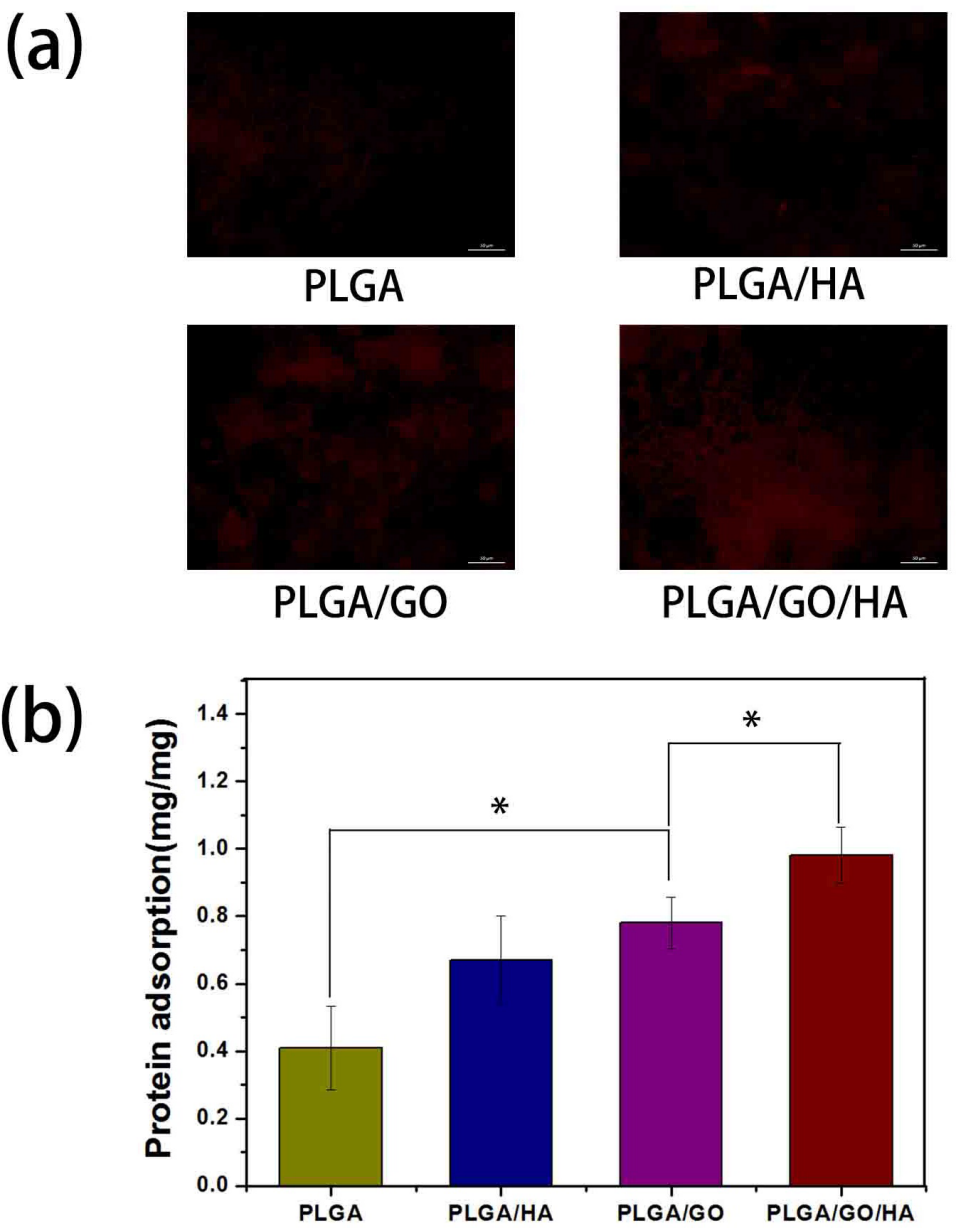
Protein adsorption on different nanofibrous matrices. (a) Fluorescence images of the Rhodamine B labelled BSA adsorption on PLGA, PLGA/HA PLGA/GO and PLGA/GO/HA; (b) The adsorption of protein onto the PLGA, PLGA/HA, PLGA/GO and PLGA/GO/HA nanofibrous matrices. (n = 5;* p < 0.05).

The adsorption of proteins onto the surface of materials is highly related to biocompatibility of the materials. Therefore, bovine serum albumin (BSA) was selected as a model protein to examine the adsorption efficiencies of synthesized nanofibrous matrices. As shown in Fig 4, the BSA adsorption was determined to be 0.67 ± 0.13 and 0.78 ± 0.13 mg on PLGA/HA and PLGA/GO respectively, which were obviously higher than that on PLGA nanofibrous matrices. It is deduced that the higher protein adsorption capacities of nanofibrous matrices might result from their improved surface properties and larger specific surface areas after the incorporation of HA and GO. Furthermore, the hydrophilicity property and rough surface morphology of nanofibrous matrices is one reason for the improvement of protein adsorption capacity. Recently, it was reported that GO has strong capabilities to adsorb various proteins, including cytochrome c, bovine serum albumin, ribonuclease A, and protein kinase A [29–31]. In this study, PLGA/GO have also exhibited higher protein adsorption capacities, nearly twice that that of pure PLGA nanofibrous matrices (p<0.05). Moreover, it has been found that the protein adsorption capacities of PLGA/GO nanofibrous matrices was slightly greater than the PLGA/HA nanofibrous matrices. Among the nanofibrous matrices, the highest protein adsorption was obtained for the PLGA/GO/HA nanofibrous matrices, nearly 1.46 and 1.25 times of adsorption rates of adsorption rates than those of PLGA/HA and PLGA/GO nanofibrous matrices (p<0.05), respectively. GO and HA showed the additive effect of improving the protein adsorption capacities of polymer materials. To directly observe the adsorbed protein on different matrices. Rhodamine B labelled BSA in PBS solution was incubated with different nanofibrous matrices for 2 h. The fluorescence images of the BSA adsorption on PLGA, PLGA/HA, PALGA/GO and PLGA/GO/HA nanofibrous matrices were also performed to determine the protein adsorption capacities of different samples. As shown in Fig 4, compared to the PLGA nanofibrous matrices, the high density of adsorbed protein was obtained on PLGA/GO and PLGA/HA nanofibrous matrices. Furthermore, PLGA/GO/HA nanofibrous matrices show the highest density of adsorbed protein, indicating that the protein adsorption capacity of PLGA nanofibrous matrices was significantly increased by blending with GO and HA. The above protein adsorption trend was consistent with the quantitative measurements.

### Mechanical properties

**Fig 5:**
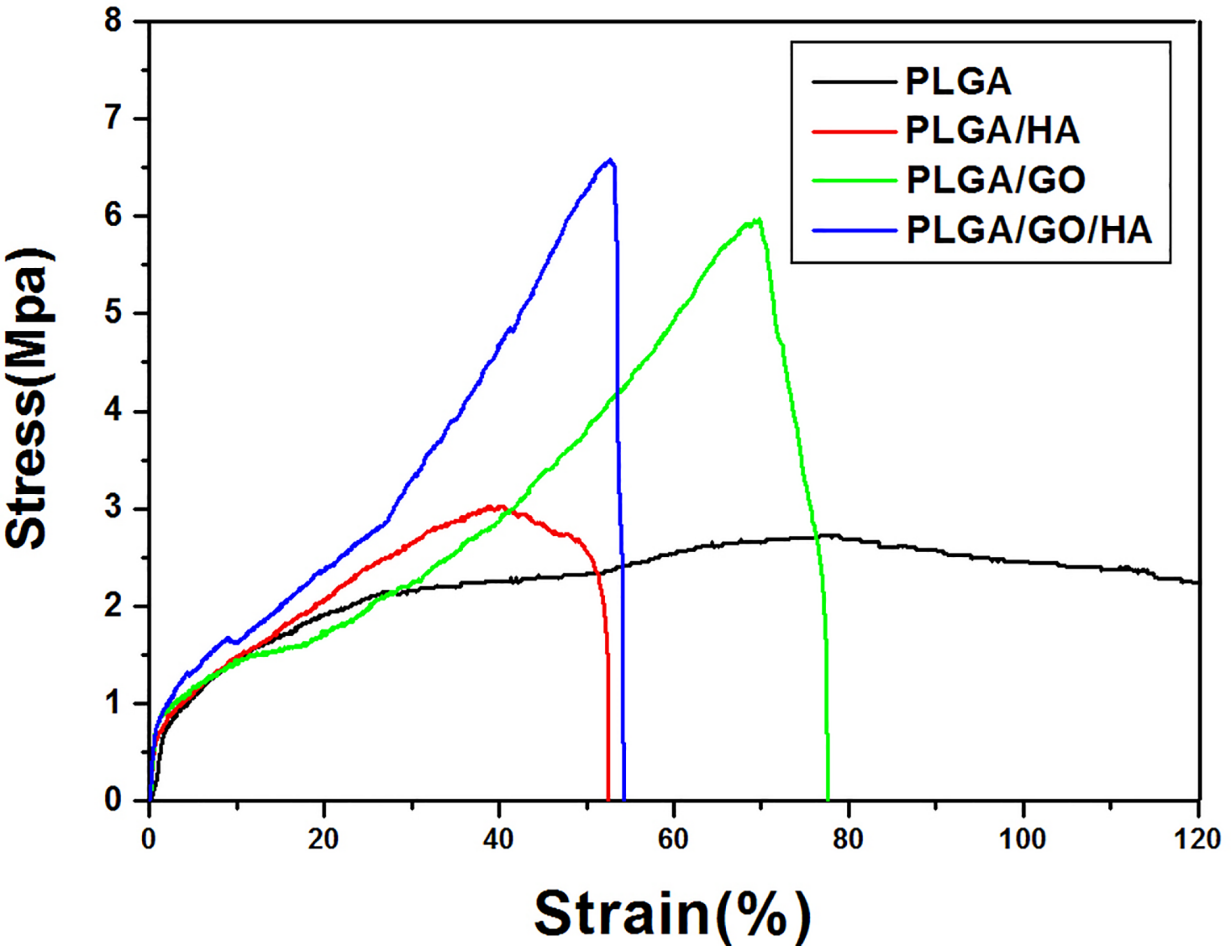
Stress–strain curves of the PLGA, PLGA/HA, PLGA/GO, and PLGA/GO/HA nanofibrous matrices were obtained under a cross-head speed of 5 mm/min. Prior to testing, 4 types of matrices were cut into a rectangular shape, 30 mm in length and 10 mm in width.

Scaffolds for tissue engineering should be mechanically robust. Therefore, maintaining the mechanical properties of the PLGA nanofibrous matrices is vital to its biomedical applications. Fig 5 shows the typical stress-strain curves of the PLGA, PLGA/HA, PLGA/GO and PLGA/GO/HA nanofibrous matrices. The tensile strength for PLGA、PLGA/HA、PLGA/GO and PLGA/GO/HA are 2.72 MPa (black line)、3.02 MPa (red line)、5.98 MPa (green line) and 6.58 MPa (blue line), respectively. Compared with the pure PLGA nanofibrous matrices, the HA/PLGA nanofibrous matrices exhibit higher tensile strength. Previous studies have demonstrated that HA can improve the mechanical properties of polymer materials to a certain extent [32, 33]. However, the lack of adhesion between the HA and PLGA matrix resulted in early failure at the HA-polymer interface, thus HA alone possesses a limited ability to improve the mechanical properties of polymer materials. When GO was added to the PLGA nanofibrous matrices, the tensile strength of nanofibrous matrices increased significantly. The GO-impregnated nanofibrous matrices have exhibited higher tensile strength, by almost 2.19-fold and 1.98-fold, respectively, than that of PLGA and PLGA/HA nanofibrous matrices. This increase may be attributed to the interfacial interaction between the oxygen-containing functional moieties of GO and the hydroxyl or amine groups of the substrates, which can improve the interfacial interactions between graphene oxide and the polymer materials [34]. These result indicate that blending PLGA/HA nanofibrous matrices with GO did have a show significant influence on improving the mechanical properties of the polymer materials, thereby making them more suitable for bone tissue regeneration.

### Cell morphology and proliferation on different nanofibrous matrices

**Fig 6:**
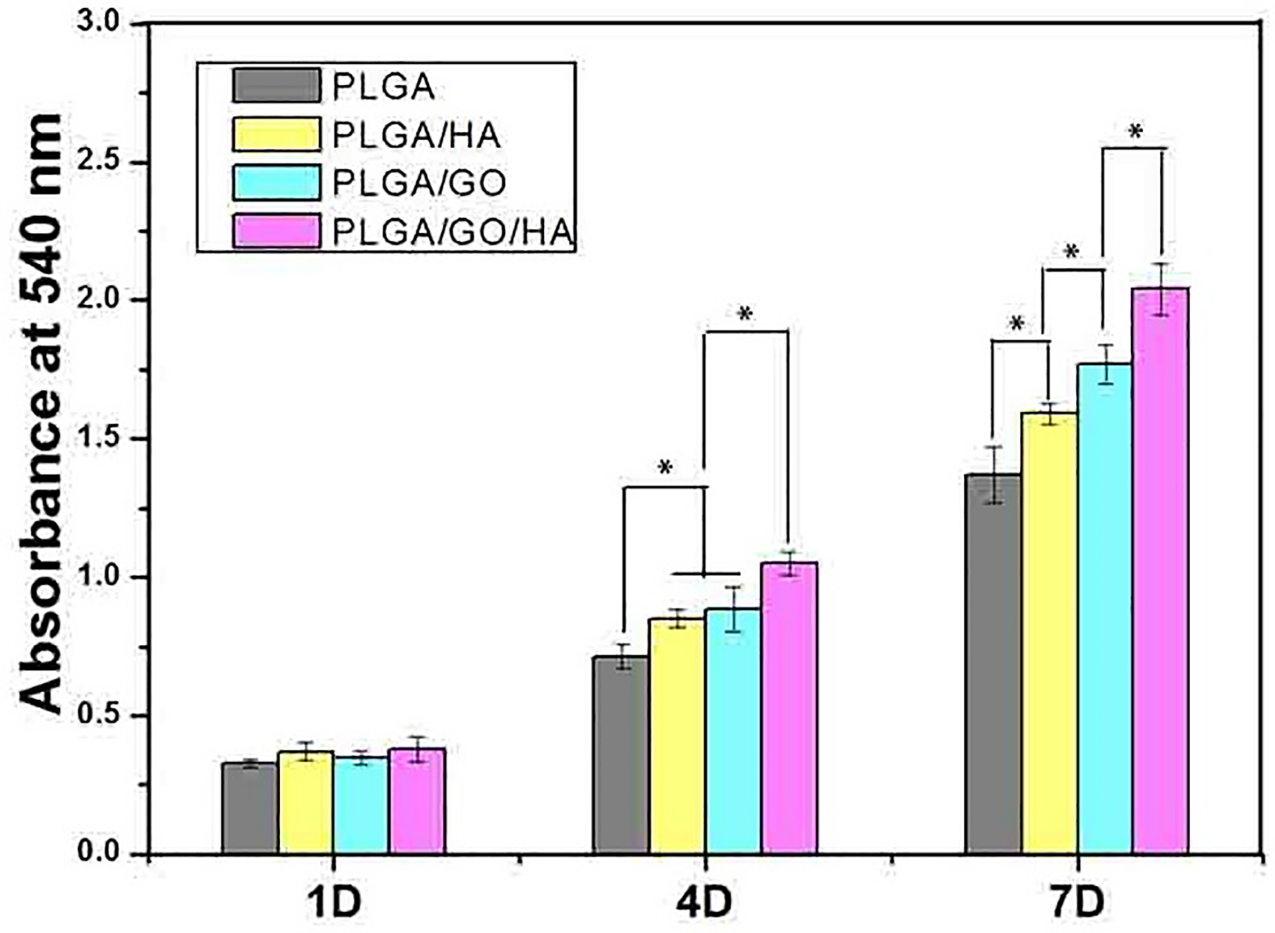
Proliferation of MC3T3-E1 cells cultured on the nanofibrous matrices for 1 to 7 days in vitro. P < 0.05, n = 4.

Enhancement of cell adhesion and proliferation on the nanofibrous matrices is a crucial factor for promoting osseointegration. The MC3T3-E1 cell adhesion and proliferation were investigated to evaluate how the added GO and HA affected the cellular metabolism. As shown in Fig 6, The MC3T3-E1 cells proliferation was monitored quantitatively via the MTT assay to measure the metabolic activity of the total population of cells at 1 d, 4 d and 7 d. The metabolic activity (OD value) of MC3T3-E1 cells on different samples increased gradually with the prolonged culture time. It was clear that the OD values of cell viability showed the lowest level on the PLGA nanofibrous matrices at 4 and 7 d. The cell proliferation was significantly greater in the PLGA/GO and PLGA/HA groups compared to PLGA at 4 and 7 days (p<0.05). Previous studies have demonstrated that 10%HA could promote the adhesion on the PLGA films, due to its rough surface after HA doping [18]. Furthermore, we found that the OD values of MC3T3-E1 cells on PLGA/GO nanofibrous matrices were observed slightly higher than the PLGA/HA at 7 d (p<0.05), which may be due to the increased protein adsorption and hydrophilicity. The highest OD values of the cells were observed on PLGA/GO/HA at 4 and 7d (p<0.05), indicating that the combination of GO and HA had a synergistic effect in terms of cell proliferation.

**Fig 7:**
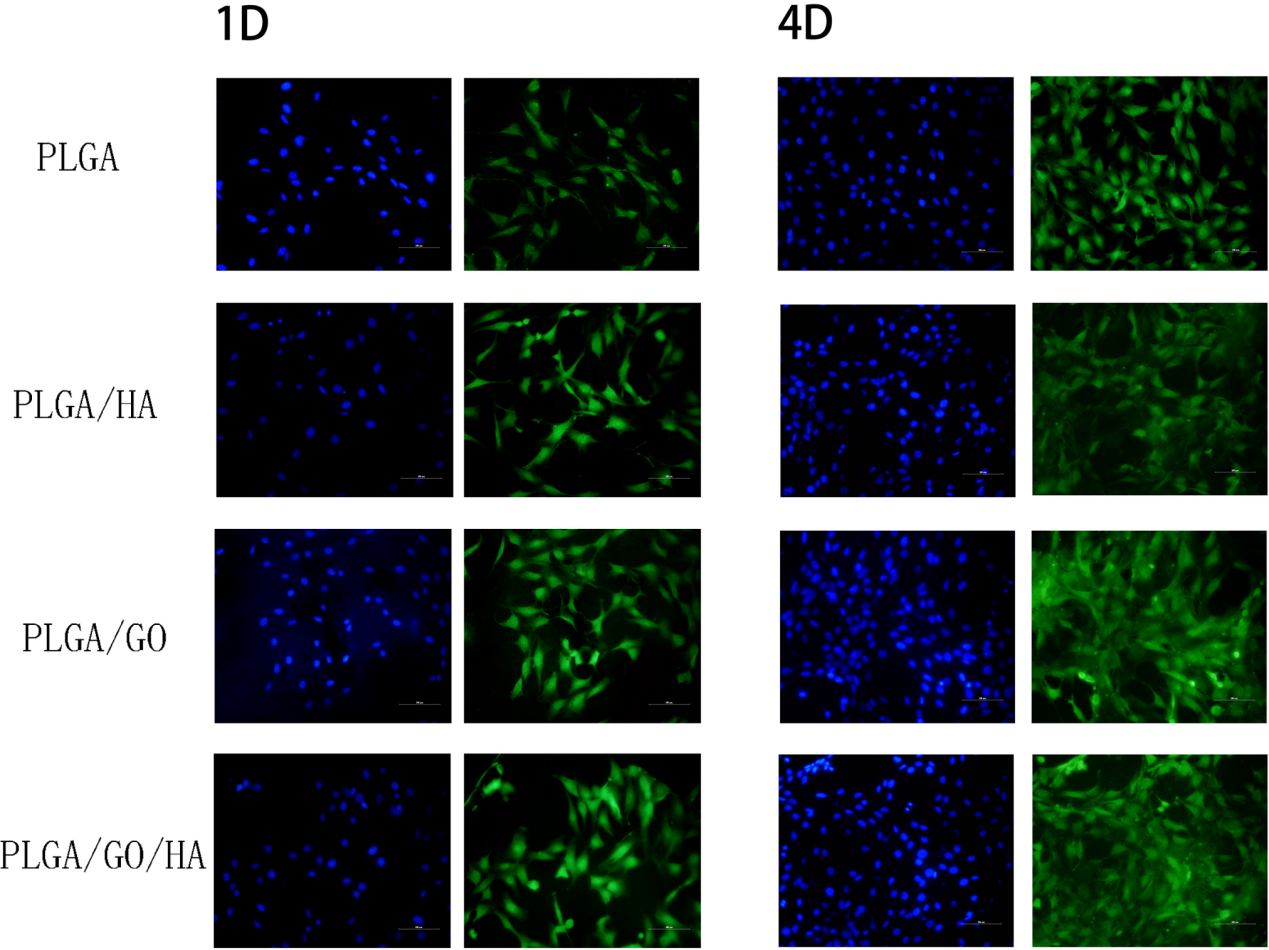
Fluorescent staining observation of MC3T3-E1 cells cultured on different nanofibrous matrices for 1 and 4 days: MC3T3-E1 cells have been cultured on nanofibrous matrices with cytoskeleton (FITC, green) and nucleus (DAPI, blue) staining. All scale bar lengths are 100 μm.

As shown in Fig 7, the morphology of the MC3T3-E1 cells grown on different nanofibrous matrices at 1 and 4 d was observed using cytoskeleton (green) and nuclei (blue) fluorescent staining. One day post-seeding, the results of a nuclear staining showed that there was no significant difference in cell number of MC3T3-E1 cells cultured on different nanofibrous matrices. However, cells in the PLGA nanofibrous matrices show a slimmer shape with less spread; in contrast, cells in the nanofibrous matrices with GO and HA demonstrate enhanced cellular spreading and a well-organized cytoskeleton. After 4 days, compared to the PLGA nanofibrous matrices, more adhered MC3T3-E1 cells were observed on PLGA/GO and PLGA/HA nanofibrous matrices. Among them, PLGA/GO/HA nanofibrous matrices exhibited the best cytoskeleton, indicating that the electrospun nanofibrous matrices containing GO and HA were beneficial for cell growth and cell–cell communication.

### ALP activity

**Fig 8:**
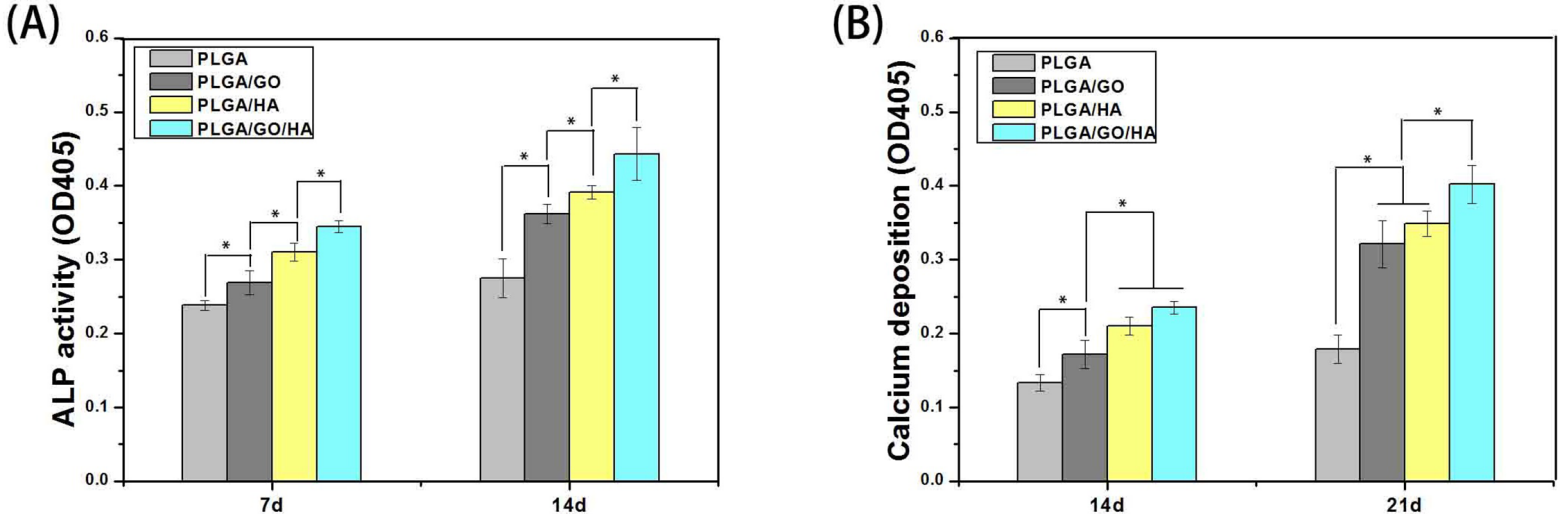
ALP activities of MC3T3-E1 cells in PLGA, PLGA/GO, PLGA/HA and PLGA/GO/HA nanofibrous matrices during 14-day in vitro culture (A); Calcium deposition after culturing in PLGA, PLGA/GO, PLGA/HA and PLGA/GO/HA nanofibrous matrices for 14 and 21 days (B). P < 0.05, n = 4.

The high reactivity of ALP represented the osteogenic differentiation of the cells. Therefore, ALP can be considered as an early interim osteoblast activity indicator. As shown in Fig 8A, the intracellular ALP activity from cells on PLGA/GO and PLGA/GO/HA nanofibrous matrices was significantly higher than that on PLGA nanofibrous matrices. Previous studies have demonstrated that GO could significantly increase the ALP expression of a variety of cells [27, 35, 36]. Compared with PLGA/GO, the ALP activity of cells in the PLGA/HA nanofibrous matrices was higher. As the primary mineral component in mature bone, HA has the excellent abilities of bone binding and osteoconductivity, and previous studies have demonstrated that HA can increase the ALP activity of cells and induce the cells towards osteogenic differentiation [19, 37]. Furthermore, when GO was associated with HA (PLGA/GO/HA), the nanofibrous matrices exhibited a stronger ability to increase the ALP activity of MC3T3-E1 cells than either PLGA/HA or PLGA/GO alone (p<0.05).

### Mineralization

**Fig 9:**
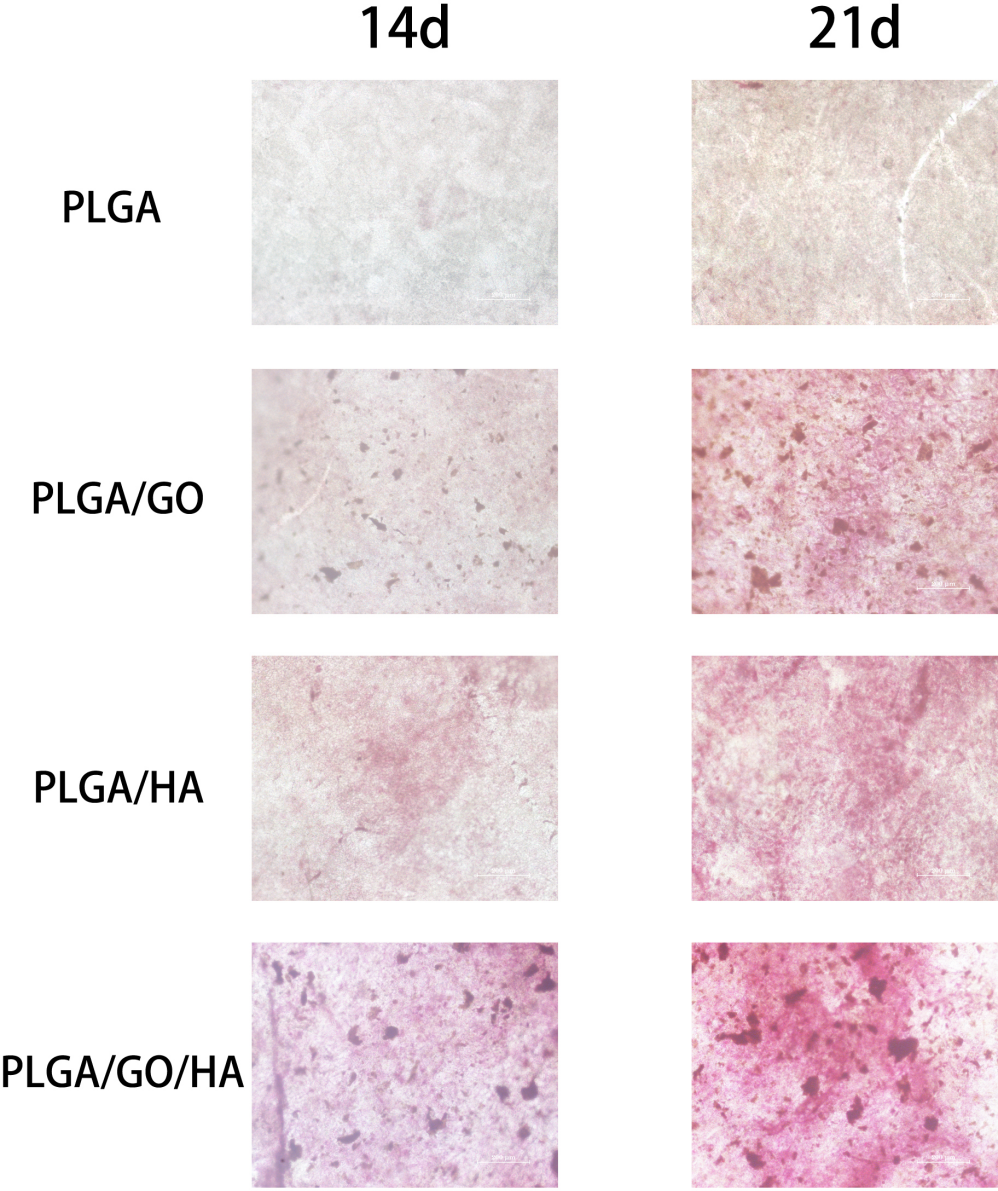
Alizarin Red staining of MC3T3-E1 cells cultured on PLGA, PLGA/GO, PLGA/HA and PLGA/GO/HA nanofibrous matrices at 14 and 21 days.

When cells differentiate on polymer materials, anionic matrix molecules will take up Ca^2+^, followed by phosphate ions and thus lead to calcification through nucleation and growth. ARS staining can bind to Ca^2+^ in mineralized ECM showing bright red stains. Therefore, we analysed the efficiency of the mineralization stage using ARS staining. As shown in Fig 9, at day 14 and 21, the Alizarin Red stain shows slight reddish dots on PLGA/GO, PLGA/HA and PLGA/GO/HA nanofibrous matrices, but almost no positive stains were found over the PLGA nanofibrous matrices. Furthermore, the noteworthy formation of calcium depositions by PLGA/GO/HA nanofibrous matrices was observed from 14 to 21 days, indicating that GO and HA synergistically induced calcium deposition in MC3T3-E1 cells. The dissolved ARS extracted from the staining plates also quantitatively demonstrated the above qualitative calcium staining pattern (Fig 8B). Interestingly, at day 21, the deposition of calcium mineral in the PLGA/GO nanofibrous matrices was similar to that in the PLGA/HA. We speculated that the excellent protein adsorption and hydrophilicity of PLGA/GO can not only promotes cell proliferation but also improve the nucleation of HA, which facilitate the late stage marker of osteogenic differentiation. Furthermore, a recent study reported that GO is able to elevate not only ALP activity but also calcium deposition in cells [36].

**Fig 10:**
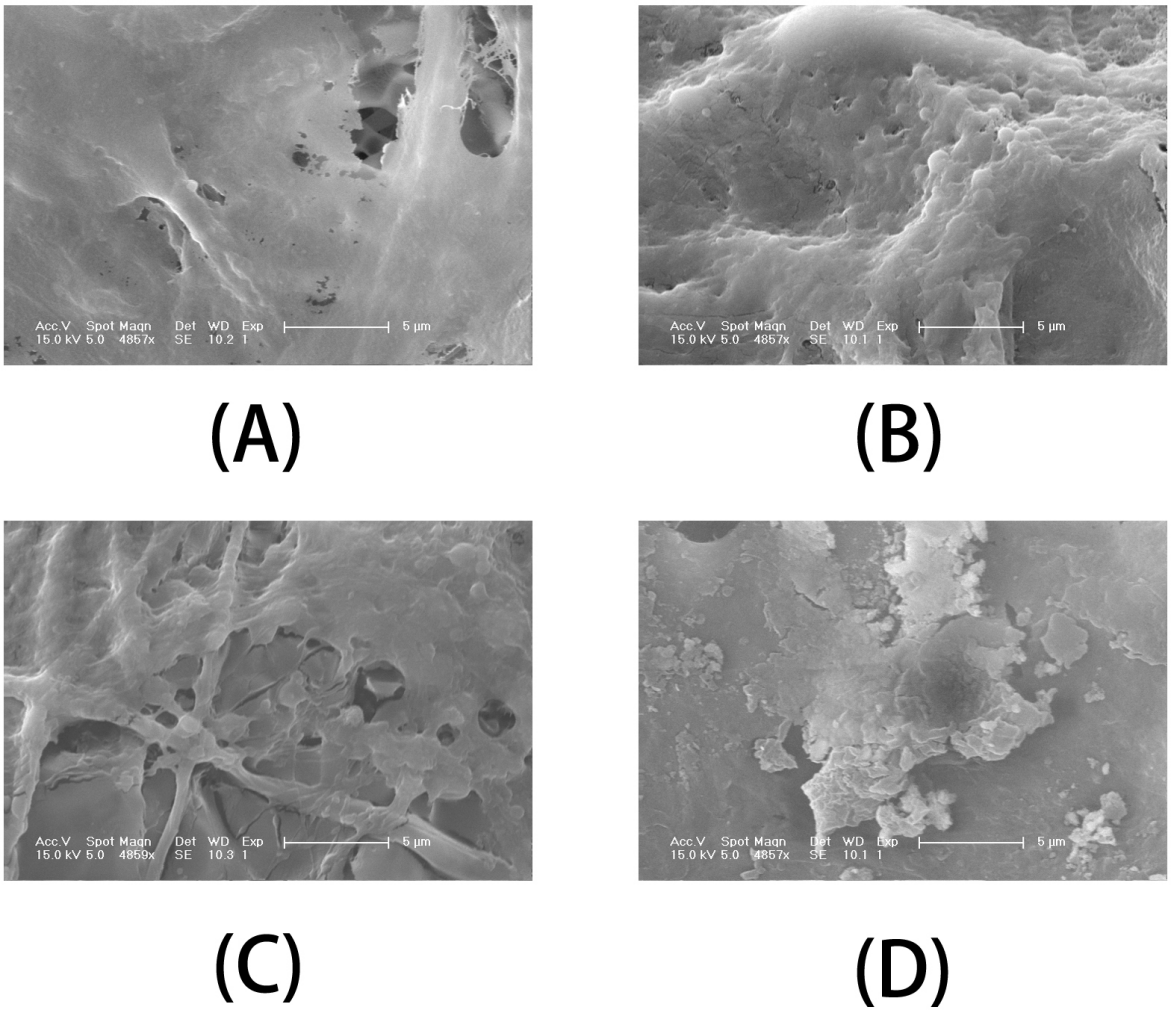
SEM images of MC3T3-E1 cells on (A) PLGA, (B) PLGA/GO, (C) PLGA/HA and (D) PLGA/GO/HA nanofibrous matrices after 21 days of mineralization.

To visualize the effect of different nanofibrous matrices on cell mineralization in cultured MC3T3-E1 cells, SEM images of the four kinds of nanofibrous matrices after 21 days incubation were acquired. Scanning electron microscopy (Fig 10) indicated that cells growing on PLGA/HA and PLGA/GO nanofibrous matrices were more densely mineralized at 21 days than those on PLGA nanofibrous matrices. Among all samples, PLGA/GO/HA nanofibrous matrices were the most densely mineralized. The SEM results also depicted the same trends observed from the ARS staining. The above results indicate that the importance of an additive effect in the mineralization process of MC3T3-E1 cells by combination of GO and HA.

### Bone-related gene expression by qRT-PCR tests

**Fig 11:**
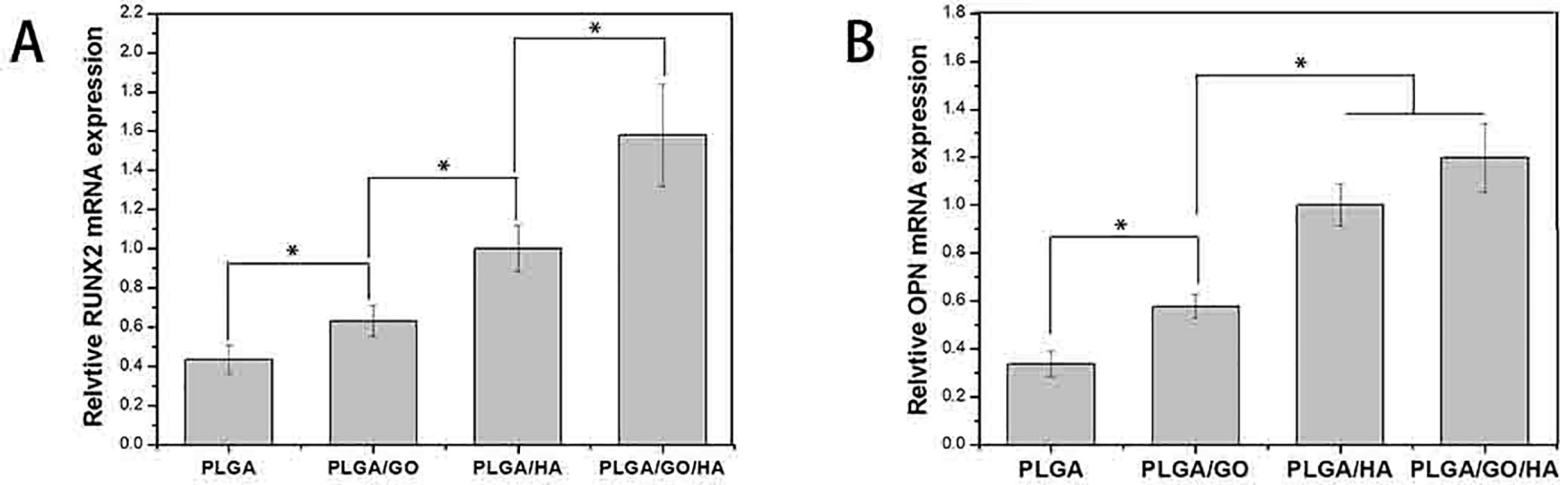
Quantitative real-time PCR analysis of osteogenesis-related gene expression of RUNX2 (A) and OPN (B) after MC3T3-E1 cells cultured for 7d. P < 0.05, n = 3.

The initialization and completion of cell functions such as differentiation are accompanied with a series of intracellular regulating gene expression. For example, RUNX2 is an early differentiation marker observed at the early stage of differentiation, while OPN expression is observed at the middle stage of differentiation. As shown in Fig 11, after 7d of culture, the expression of RUNX-2 was elevated to a higher level on the PLGA/GO and PLGA/HA nanofibrous matrices compared with the PLGA, and the expression of OPN was at a similar level for all the nanofibrous matrices. It is reported that GO could promote the osteogenic differentiation of MC3T3-E1 cells by increasing COL-I, BSP, Runx2 and OCN expression [38, 39]. Moreover, HA, as a normal constituent of bone, could promote cells osteogenic differentiation and matrix synthesis. Among all the groups, PLGA/GO/HA nanofibrous matrices showed the highest expression levels of RUNX2 and OPN, nearly 2.4-fold for RUNX2 and 3.9-fold for OPN higher than PLGA nanofibrous matrices. The findings in this study showed that the PLGA blend with GO and HA can enhanced the osteodifferentiation of MC3T3-E1 cells.

In recent years, biodegradable polymers such PLGA have been widely used to prepare bone tissue engineering scaffolds due to their versatile properties, but their hydrophobic properties and lack of osteogenic activity seriously limit their biological applications [11]. A major aim of this work was to improve the cell adhesion and osteogenic activity of bone implants for osseointegration. Hydroxyapatite is a major inorganic component of natural bone, and has the abilities of bone binding and osteoconductivity [18, 40]. The combination of HA and polymer materials can be expected to attain the optimum scaffold for bone tissue engineering. In this study, the protein adsorption and cell proliferation of nanofibrous matrices were significantly improved by blending with HA due to the increase of the specific surface areas, and surface roughness. More importantly, the levels of ALP activity, calcium deposition and osteogenic gene expression of MC3T3-E1 cells were increased obviously by the incorporation of HA. However, there are two most problematic issues in manufacturing PLGA/HA composite, HA by itself possesses limited osteoinductive ability, and its mechanical properties cause it to be brittle with a poor fatigue resistance [19, 20]. Therefore, it is necessary to tune the mechanical properties, biocompatibility, and osteoinductive ability of PLGA/HA composites in some way.

A growing number of studies suggested that GO can act as both an effective reinforcement and a bioactivator of polymer materials to manipulate bone cells behaviour. Dai et al. indicated that GO could promote the proliferation and differentiation of mouse mesenchymal stem cells [27]. Zhao et al. found that the poor mechanical properties of PLGA-collagen nanofibrous matrices can be effectively improved by blending with GO [41]. In this study, GO was chosen for use in PLGA/HA nanofibrous matrices since the carboxylic acid and carbonyl groups on the surfaces of the GO nanosheets were expected to generate improved bioactivity and mechanical properties of nanofibrous matrices. When GO was introduced to PLGA/HA nanofibrous matrices, the mechanical properties, ALP activity, calcium deposition and osteogenic gene expression became obviously stronger than those of the other groups, indicating that the shortcomings of PLGA/HA composites could be overcome by adding GO. More importantly, we found that the combination of GO and HA had a synergistic effect in terms of hydrophilicity, protein adsorption and osteogenic induction. Thus, we herein demonstrate that PLGA/GO/HA nanofibrous matrices are a promising bone tissues scaffold for bone regeneration. In future work, the PLGA/GO/HA nanofibrous matrices will be further investigated with in vivo experiments.

## Conclusions

In this study, nanocomposite nanofibres of PLGA embedded with GO and HA were prepared by electrospinning, and a detailed characterization of the PLGA/GO/HA nanofibrous matrices, including mechanical properties, hydrophilicity, and protein adsorption capacity was performed. Our results show that the protein adsorption and hydrophilicity of the nanofibrous matrices were significantly improved by blending with GO and HA. An interesting observation made throughout the experiment is that the incorporation of GO nanosheets led to the effective enhancement of their mechanical properties compared to those of PLGA and PLGA/GO/HA nanofibrous matrices. In vitro studies including MTT assays, fluorescence staining, ALP activity, calcium deposition, qRT-PCR analysis indicated that the significantly high cellular activities and osteogenic markers expression on PLGA/GO/HA nanofibrous, suggesting that the GO and HA have the additive effect of promoting proliferation and osteogenic differentiation of MC3T3-E1 cells. Above all, our results indicated that PLGA/GO/HA matrices exhibit good biocompatibility, mechanical properties and improve the osteoblast cell functions. Therefore, we envision that PLGA/GO/HA nanofibrous matrices have great potential for the development of biodegradable bone scaffolds and implants. However, further studies in vivo are still needed to evaluate their efficacy in promoting bone regeneration.
